# Impact of social stigma on the process of obtaining informed consent for genetic research on podoconiosis: a qualitative study

**DOI:** 10.1186/1472-6939-10-13

**Published:** 2009-08-22

**Authors:** Fasil Tekola, Susan Bull, Bobbie Farsides, Melanie J Newport, Adebowale Adeyemo, Charles N Rotimi, Gail Davey

**Affiliations:** 1 School of Public Health, Addis Ababa University, Addis Ababa, Ethiopia; 2 Brighton and Sussex Medical School, Falmer, Sussex, UK; 3 Centre for Research on Genomics and Global Health, National Human Genome Research Institute, National Institutes of Health, USA; 4 The Ethox Center, Division of Public Health and Primary Care, University of Oxford, UK

## Abstract

**Background:**

The consent process for a genetic study is challenging when the research is conducted in a group stigmatized because of beliefs that the disease is familial. Podoconiosis, also known as 'mossy foot', is an example of such a disease. It is a condition resulting in swelling of the lower legs among people exposed to red clay soil. It is a very stigmatizing problem in endemic areas of Ethiopia because of the widely held opinion that the disease runs in families and is untreatable. The aim of this study was to explore the impact of social stigma on the process of obtaining consent for a study on the genetics of podoconiosis in Southern Ethiopia.

**Methods:**

We adapted a rapid assessment tool validated in The Gambia. The methodology was qualitative involving focus-group discussions (n = 4) and in-depth interviews (n = 25) with community members, fieldworkers, researchers and staff of the Mossy Foot Treatment and Prevention Association (MFTPA) working on prevention and treatment of podoconiosis.

**Results:**

We found that patients were afraid of participation in a genetic study for fear the study might aggravate stigmatization by publicizing the familial nature of the disease. The MFTPA was also concerned that discussion about the familial nature of podoconiosis would disappoint patients and would threaten the trust they have in the organization. In addition, participants of the rapid assessment stressed that the genetic study should be approved at family level before prospective participants are approached for consent. Based on this feedback, we developed and implemented a consent process involving community consensus and education of fieldworkers, community members and health workers. In addition, we utilized the experience and established trust of the MFTPA to diminish the perceived risk.

**Conclusion:**

The study showed that the consent process developed based on issues highlighted in the rapid assessment facilitated recruitment of participants and increased their confidence that the genetic research would not fuel stigma. Therefore, investigators must seek to assess and address risks of research from prospective participants' perspectives. This involves understanding the issues in the society, the culture, community dialogues and developing a consent process that takes all these into consideration.

## Background

Current national and international guidelines and regulations on research ethics stress the importance of consent as a requirement for conducting ethically sound research. The assumptions underlying the practice of informed consent include that decisions about research participation should be both appropriately informed and voluntary, and be made by someone competent to reach such decisions [1,2].

Currently, there is widespread discussion and debate about some aspects of informed consent. Among these issues are the role of social and cultural factors in communicating research information and in the style of decision-making about research participation; whether informed consent should be obtained from all study subjects; and whether one standard consent form should be used [3-8].

Additional issues have to be considered when genetic research is conducted. Studies showed that individual-based informed consent model may not be fully applicable to genetic family studies in which the family is used as the unit of analysis [9,10]. In family-based genetic studies, individual participants are linked to other family members through blood relationships, and family members may become part of the study without their consent since the results of the study may affect the whole family [11].

The consent process for genetic research becomes more complex when research is conducted among a group that is stigmatized because of the genetic nature of the disease under investigation. Podoconiosis is an example of such a disease. Podoconiosis (non-filarial elephantiasis) is a chronic geochemical disease caused by long-term exposure of feet to red clay soils of volcanic origin resulting in progressive swelling of the lower legs. A recent genetic study that enrolled 59 multi-generational and multiply affected families presented evidence for a genetic basis to podoconiosis. The sibling recurrence risk ratio was calculated to be 5.07, and heritability of podoconiosis with proband ascertainment was estimated to be 62.9% (SE 0.069, p = 1 × 10^-7^). Segregation analysis showed the most parsimonious model to be that of an autosomal co-dominant major gene with age and foot wear as significant environmental covariates [12]. It affects more than 5% of the population in the Wolaita zone of southern Ethiopia. According to a survey, in 2002 there were at least 81,000 podoconiosis patients in this zone that has an estimated population of 1.6 million making the weighted zonal prevalence 5.5% [13]. The disease affects the productive segment of the population and results in an estimated loss of 1.6 million US dollars every year in the Wolaita zone [14]. A recent study indicated that 55.8% of community respondents in the zone had at least one stigmatizing attitude towards podoconiosis patients [15]. The main preventive measure for the disease is wearing protective shoes and washing feet regularly since early childhood. In addition, personal hygiene, wearing protective boots and elastic bandages, and elevation help reverse the swelling in the earlier stages. Even if there are effective preventive measures against podoconiosis, public health intervention programs in Ethiopia target their resources on health problems like TB, HIV/AIDS and malaria. The genetic study on podoconiosis opens the door for targeting available scarce resources for education programs, provision of shoes, socks and water supply to susceptible families that are most at risk of having the disease.

People affected by podoconiosis in Wolaita zone are cared for primarily by the Mossy Foot Treatment and Prevention Association (MFTPA). The MFTPA is a local non-governmental organization involved in prevention, treatment and control of podoconiosis, and economic rehabilitation of affected people through micro-financing activities. It has established long standing relationships with the podoconiosis community in Wolaita zone. The MFTPA has developed strategies that facilitate trust among affected people who are sceptical of being stigmatized by the unaffected. A key component of these strategies involves the deployment of treated podoconiosis patients back into their communities after training as health educators by the MFTPA. These fieldworkers serve as role models for the remaining podoconiosis-affected community, and are socially accepted because they belong to the community. Their activities include health education, treatment, recruitment and counselling of patients through home visits; creating networks with governmental sectors; and educating non-affected members of the population. Currently, the organization treats more than 30,000 patients per year through 15 outreach clinics. The treatment provided by the organization is simple and effective in reversing progression of early stage disease. A recent study shows that there is an improvement in the quality of life of patients treated under the MFTPA [16]. Furthermore, many patients resume productivity after treatment. These achievements contributed to the reduction of stigma because unaffected members of the community observed that the disease can be treated (Interview with MFTPA staff).

Because of its high prevalence, and social, psychological and economic impact, podoconiosis is a health problem of public health importance in endemic areas. Affected patients and families are illiterate and extremely poor. In addition, the disease clusters within families making affected families targets for social stigmatization. Affected persons suffer from various forms of stigma manifested during marital and social events, employment, and schooling [17]. The stigma linked with having podoconiosis is so deep rooted that it plays a central role in many aspects of life of the affected. The proposed genetic study looks at the familial component of the disease that is the main cause of stigmatization, and may entail risk of increasing stigma. As a consequence, there is need to identify a sensitive way to approach this population that has been historically stigmatized and minimise potential increase in stigma as a result of conducting the genetic study. Obtaining consent from podoconiosis patients and enrolling them in genetic research entails prior understanding of how the prevailing stigma shapes their fears and concerns, and the way they make choices. This process of understanding allows researchers to design appropriate consent processes for the local context. The rapid assessment was designed to provide information about this and findings were used to inform the design of the consent process for the genetic study.

In this paper, we describe the development, implementation and interpretation of a rapid assessment in identifying the manifestations of stigma in podoconiosis affected families; the role of stigma in decision-making about participation in family-based genetic research; and the interaction between stigma and participants' trust in investigators and local institutions in making decisions for research participation. The information gathered facilitated the design and implementation of the consent process subsequently used in an international collaborative study on the genetic basis of susceptibility to podoconiosis using data from affected sibling pairs, their biological parents and unaffected controls.

## Methods

### Study area and design

The study was conducted in Wolaita zone which is located in Southern Ethiopia covering a total area of 4541 sq. kms. The methodology was entirely qualitative involving focus group discussions (FGDs) and in-depth interviews (IDIs). We used qualitative methodology because the primary purpose of the study is to explore the views, perspectives, experiences, preferences and concerns of the study participants. The study was conducted using semi-structured interview guides adapted from a rapid assessment method validated in The Gambia [18]. The study instruments included topics on perception of the community about podoconiosis and manifestations of stigma associated with the disease; issues and concerns of the MFTPA about genetic research on podoconiosis; and how stigma impacts motivation and decision making pattern of participants for genetic research. Pilot testing of the instruments was done with one researcher in the United Kingdom and a fieldworker and community member in Wolaita.

### Sampling

Generally, sampling was purposive based on pre-defined inclusion criteria for enrolling participants. IDIs and FGDs were conducted until no new relevant ideas emerged from further interviews or discussions. The study targeted four groups of participants. The first group incorporated scientists and researchers that had experience working in Wolaita Zone on genetics or other biomedical studies. IDIs were conducted with four scientists and researchers in this phase of the study. The second group included trained MFTPA fieldworkers. IDIs were conducted with three social and counselling workers and four health workers. The third group involved IDIs with (i) two individuals involved in the administration and coordination of the activities of the MFTPA, (ii) two heads of *kebele *(the smallest administrative unit/village in Ethiopia) and (iii) two community leaders. The fourth group comprised community residents of both sexes and included patients and healthy subjects. In total 32 community members participated, 8 in IDIs and 24 in 4 FGDs.

### Data collection

With the exception of the community interviews, data were collected by one of the principal investigators. An experienced Masters in Public Health graduate who speaks Wolaitigna (the local language) did the IDIs and moderated the FGDs with community members. Before conducting the IDIs and FGDs, he was trained about the purpose of the study, the data collection instruments and interviewing techniques.

### Data analysis

Audiotapes were transcribed anonymously, and interviews conducted in Amharic and Wolaitigna were translated into English. During translation, we tried to keep phrases and words as in the spoken language. However, we acknowledge that some information carried by culturally flavoured phrases could be lost when expressions spoken in the local languages were translated into written English style. Data were imported into OpenCode software v.2.1 (a freely available computer program for managing and analyzing text data) [19]. Open coding was used to identify themes that were developed into conceptual categories. Data were iteratively examined to identify additional themes. The issues that arose fell into the following primary thematic domains: stigma associated with podoconiosis; issues related to genetic research; role of stigma in risk perception, motivation and decision making for participation in genetic research; and place of trust in the consent process.

### Ethical considerations

The ethical review board of the Faculty of Medicine, Addis Ababa University and the Ethiopian Science and Technology Agency approved the study. Informed verbal consent was obtained from the study participants before conducting the interviews and discussions. The consent process was audio-taped for record purposes. Discussions and interviews were conducted in settings that ensure participants' privacy.

## Results

Overall, 46 individuals (27 males and 19 females) participated in the study. Half of the community FGD participants, half of the researchers and one of the fieldworkers interviewed were females. All interviewees from MFTPA management and *kebele *offices were males. The age of the respondents ranged between 23 and 70 years. The educational status of the fieldworkers ranged between early secondary level and college level education. Most of the community interviewees had had no formal education.

### Social stigma associated with podoconiosis

There were considerable variations among community members' understanding of the causes of podoconiosis including genetic susceptibility, snakebite, direct contact of feet with clay soil, contagious, curses from God and poor nutrition. Other less commonly mentioned reasons were injuries (e.g., cut with axe), exposure to condensation, washing feet in hot weather and the evil eye. Genetic susceptibility was the most frequently reported cause of podoconiosis and this belief was observed to play a central role in stigmatization of podoconiosis affected families.

Participants from the community unanimously described podoconiosis as a condition that results in debility, poor self-confidence, social isolation and poverty. The local vernacular term for podoconiosis is *gediya kita*, which means leg swelling and is used as a derogatory term. Stigma against podoconiosis patients is multi-faceted and extensive. Manifestations of the stigma commonly mentioned by participants were (1) unwillingness to marry a diseased person or anyone from a podoconiosis-affected family; (2) shunning of patients and family members; (3) avoiding physical contact with patients; (4) excluding patients from social events like weddings and funerals; (5) spitting on patients; (6) pinching nose when walking past patients at a distance; (7) unwillingness of classmates to sit with patients at the same desk in school; and (8) unwillingness of unaffected family members to approach an affected household member. Consequentially, patients feel guilty, hide and isolate themselves from the rest of the community members. Most patients described it as 'the worst disease' mainly due to its negative social consequences and absence of an effective cure at the advanced stage.

In my opinion this is the worst of all diseases. It is better to die than catching this disease because it keeps you at home and you starve and thirst while you are alive. [Male participant]

It breaks the social bond even with loving friends. [Female participant]

They see people from leg to head, not up down. A healthy person never likes to marry a podoconiosis patient. An unaffected lady never likes to marry a podoconiosis-affected man. [Fieldworker]

We also found that stigma impacts treatment seeking behaviour. When asked why some affected patients do not go to the MFTPA treatment centres, participants said either that they despaired about their problem or that they feared the discriminatory label given to them as 'foot patients' when other people see them going to the centre. In addition, they mentioned that some young patients do not like to wear the distinctive and rather clumsy shoes locally produced by the MFTPA because they mark them out as having podoconiosis and target them for social stigma.

We pray that people defeat their fears. Some we know them heavily affected, but do not like to come because they become afraid of others. They say, 'what will others say about me?' People know they are patients or there are rumours their children's legs for example are swollen. I cannot understand why they are afraid. [Male participant]

The shoes are also known by everyone, they say 'their shoes'. Some pay their money to buy the shoes, they not wear them in public places because people say he is a patient. [Male participant]

As a consequence, podoconiosis patients do not like to share information about their health problem with non-affected people for fear it will fuel the stigma. Patients need time to establish trust with researchers to ensure the information they provide is kept confidential. As would be expected, fieldworkers observed that this lack of trust represents a potential barrier to obtaining consent and genuine information from podoconiosis patients.

I am not happy when other people know about my disease. Why should I tell people who undermine? Why should I make those people laugh at me? I tell people like you because I can be helped and cured. [Female participant]

Yes, I mind people knowing about my health problem because people insult and stigmatize us. I would not have given you this interview if there had been someone else. [Male participant]

It is only stigma and discrimination that we get from them. Therefore, I will tell people like you if I have to tell. I am not happy telling other people. [Female participant]

Because of the long-term effects of the diseasein resulting in stigma and isolation of patients from the rest of the community, affected people hesitate to confidently talk, discuss and disclose their private issues. This is one thing that we are usually in difficulty. [MFTPA staff]

Patients demonstrated great trust in the MFTPA staff and preferred to be approached by MFTPA staff if information about their condition was sought. This is because MFTPA fieldworkers are former podoconiosis patients and, more importantly, they fully understood the social implications if confidentiality was not maintained during treatment or research.

They [MFTPA fieldworkers] are giving us a good service, they give us education, they monitor our progress, they give us shoes and under God they give us treatment. They also keep our secret that is why right now I am telling you the truth. [Male participant]

### Established trust in the MFTPA and concerns of genetic research

The MFTPA was greatly trusted by podoconiosis patients because of the services it provided and the strategy it followed. The MFTPA mainly targeted interventions against environmental triggers for the disease, primarily prevention of long term exposure of feet to irritant clay soil. Patients said the treatment provided improved their health status and positively changed the attitude of others towards podoconiosis patients, and hence contributed to reduction of stigma. They also had confidence in the fieldworkers because they are former podoconiosis patients and are trusted that they work for the good of those affected.

People used to believe we are hopeless, and the disease cannot be prevented or treated. They never like to marry our women even if they are beautiful. This organization is a gift from God. Now things are changing. For example, [name of a female fieldworker] is married to non-affected man. She earns good salary, even better than I do. They have two children and live a happy life. There are also other stories like this. [Fieldworker]

Thanks to God! Previously even my friend was spitting on me when my legs were swollen and smelling bad, but now I am equal with other friends of mine. I can say MFTPA is sent to me from God. Look at my leg it is good now; previously it was deformed; it reached this stage with MFTPA drugs and shoes. [Female participant]

However, the MFTPA had not mentioned familial factors in its communication with patients prior to the rapid assessment being conducted. MFTPA staff were concerned that raising the issue of genetics as a factor in the causation of podoconiosis might erode the Association's relationship with the podoconiosis community. They said that overemphasis on the genetic component of podoconiosis might disappoint patients and families who had already been targets of stigmatization in the community because of the prevailing belief that the disease is familial. They said that patients would be comfortable if we simply tell them, 'we do this study to understand more about the disease', and they suggested not mentioning specifically that we were conducting genetic research.

It is difficult to talk about genetics among podoconiosis patients. Most of them do not like to hear that the disease is familial. And we do not focus on that part. We teach them that exposure to the soil is the cause of the problem. They have seen that protection of feet from the soil is preventive and helps in treating early stage disease. They accepted this, and talking about genetics may confuse them. That is what unaffected people say, and our patients do not like to hear it from us. [MFTPA staff]

### Issues related to genetic research

In Wolaita, people are aware, to some extent, of what genetic predisposition means. However, there is no equivalent local term that precisely translates the word "genetics". The community commonly uses expressions like '*dabuwan adhiya harigiya*' (passed from parents and grandparents),'have it in the family', 'blood', 'bone', among others when they refer to genetic causation.

We found that most patients believe that podoconiosis runs in families. However, they do not have a clear understanding of patterns of occurrence of inherited diseases. They think that if a disease is genetic, it must be manifest in every member of the household, and should appear in every generation of the family. Any deviation from this (which is not the real heritability pattern of podoconiosis) is thought to cast doubt on the heritability (i.e., genetic nature) of the condition as expressed below:

This disease is not familial, if it was familial why my mother and I suffer from the disease while her parents were free of the disease. We probably acquired it due to lack of care for our feet. That is the way the disease catches one self. [Male participant]

Now, it is difficult to say it is familial. There is no one from either side of my parents; it was sudden when it caught me. [Male participant]

Despite believing that podoconiosis is familial, the burden of the stigma associated with the disease makes many patients deny the heritability of the disease and pretend that the disease is not present in their bloodline. Some patients were irritated when the issue of genetics was raised in discussions about podoconiosis. This response is understandable given the fact that in Wolaita, as in most parts of Ethiopia, family (bloodline relationship) has a strong social value. If a family is thought to have some factor that has negative social value, the 'blood' (or gene) of that family is considered 'unclean' (or susceptible) and thus, for example, ineligible for marriage.

Researchers who participated in this study also expressed their concern that some patients may be hesitant to participate in genetic studies for fear it would fuel stigma against them. Fieldworkers also said that even though families are willing to participate, they could under-report occurrence of the disease in their family line.

We know families with more than five affected members. Even such families do not like to hear that the disease is familial. This is partly because people who do not have the problem attribute the disease to familial factors and stigmatize them. [Fieldworker]

A solution suggested for this was selective recruitment for the genetic study of people thought to be genuine in providing such sensitive data, involving MFTPA fieldworkers and cross-checking information gained from a family with that obtained from other people who know the family without compromising the confidentiality and privacy of study participants.

Let alone this problem which is regarded as taboo in social life, when we mobilize them for less sensitive issues like income generating schemes, we talk to them repeatedly. It is usually after such repetitions and after convincing them that we will be able to touch their heart. [MFTPA staff]

A person who was leading a miserable life for a long period....one that was being discriminated against would definitely need longer to trust people and provide genuine personal information to researchers. [MFTPA staff]

### Role of stigma in risk perception and motivation for participating

We found stigma to be of central importance to participants in evaluating both the risk and long term benefit of participation in genetic research. Participants appreciated that we proposed to collect a saliva sample and that the procedure entailed no physical risk. The risk that had considerable weight was the perceived negative social outcome of the research. Patients were concerned that the research might publicize podoconiosis as a familial condition and would aggravate the stigma by labelling children of affected families as 'at-risk'. This was considered to have potentially undesirable outcomes for their self-image and perceived ability to shape their future. Fieldworkers stressed that the feared social risks should be addressed by the investigators before people are asked to consent to participate in the study. Researchers suggested addressing these concerns through a consultative dialogue between researchers and the community.

Okay, let me say my family, my neighbours and others coming here for treatment participated in your study. I am very old and will no longer worry about myself. If you say the disease is familial, my daughters will be worried day and night. Who will marry them? Everyone becomes afraid that the disease will catch her one day. Do you have solution for us? [Female participant]

Stigma also shaped the motivation of participants and their expectations of the long-term social outcome of the genetic research. Some wondered whether the research might restore their social position either by proving that podoconiosis is not familial or by providing means of developing drugs that cut transmission along generations. Provided the social risks were addressed, most patients were willing to participate in the genetic study even when we explained that participation would have no immediate benefit. Moreover, many patients were interested to know why there were variations among families in the pattern of occurrence of podoconiosis. Some participants commented that whereas the 'nodular' form of the disease seemed to run in families, the 'water bag' form did not [20].

In contrast, a member of the MFTPA staff argued that podoconiosis patients were keen to get an immediate solution for their long-lived problem and do away with the social stigma. He said this might constitute a barrier to genetic research which would only benefit society in the longer term.

Because these patients have been devoid of treatment for many years, if we tell them that our research has no immediate therapeutic benefit, it will discourage them from participating because they will despair and associate that with neglect of their problem.....we should not discourage them that the [proposed genetic] research has no immediate benefits. We should teach them that every good medication starts from research. If they are told that research helps to improve treatment, then they will be interested. [MFTPA staff]

### Impact of stigma on decision making about genetic research participation

Participants of the rapid assessment agreed that the appropriate design of the consent process, and the patterns of decision-making about participation at community, family and individual levels would depend on the type of study. Most participants said that patients are usually free to make their own decisions about participation in research. However, in relation to genetic research on podoconiosis, most participants suggested involving the head of the family, or the family as a whole in the consent process. Because of the prevailing stigma attached to a podoconiosis affected family, they (by implication) preferred that the ownership of every sample for genetic study should belong to the whole family. They were particularly sensitive about disclosing information regarding familial affection by podoconiosis. As a result, most participants expressed interest in consulting their relatives and returning with ideas before consenting. We found that these views did not differ depending on the gender of the patient.

Usually, they cooperate when they are asked for research in group by fieldworkers, but when it comes to decision to such [genetic] research, they would be interested if they are given the opportunity to consult their family. [Fieldworker]

Tendency to consult a husband/wife or any other person does not mean that there is influence in making decision. In this regard the concept of individual consent is respected... It is just a norm in the context of the study area, and that should be respected. [Researcher]

For routine research, I am the one who gives permission for all members of the family. In this case it involves giving information about every household member and descendants. Therefore, we like to discuss amongst ourselves whether or not to disclose family secret. [Male participant, head of household]

Usually, for research that involves any family member, particularly children, the father gives permission, as males are usually head of households. However, your study is sensitive. It supposes familial transmission within a family. If I decide on behalf of my child, she may be disappointed one day when she grows old if the study touches the pride of her family. My older parents, some alive, should be consulted before we participate in a study that talks about the whole family tree. [Female participant]

## Discussion

### Risk perception and trust

The study showed that the stigma associated with podoconiosis is serious and plays a considerable role in the consent process of genetic research on podoconiosis. Participants paid close attention to the potential social risks of genetic research and thought that the study might intensify the existing stigma. Participants were not only concerned about the impact of participation on their family, but about its overall impact on the larger podoconiosis community.

Researchers argue that the risks of some types of research can accrue to members of groups beyond the direct participants [21,22]. In agreement with findings that suggest that past negative experiences are easily triggered [23], our findings showed that fear of stigma and discrimination in the podoconiosis community was triggered by the idea of the proposed genetic research. As a result, managing perceived and expected social risks would be important for our genetic study, and is also relevant to the effectiveness of large science programs such as genomics [22].

Even though community level information provision (discussed below) helped lessen the perceived risks of the study, participants mainly relied on their relationship with the MFTPA whether to trust that the information provided about the proposed genetic study was genuine and that the study would be non-stigmatizing. Other studies have also shown that trust is an important factor for enrolment in research [7,21,24,25]. It is essential that potential participants trust information provided about systems to protect confidentiality, freedom to withdraw and alternative options for those who refuse [26].

In addition, our study showed that fear of stigmatization might limit the tendency of participants to provide genuine information about the disease status of other family members if they doubted the trustworthiness of the researchers. This corroborates a study that shows that trust affects participants' willingness to enrol in studies, and their tendency to tell the truth after joining studies [26].

### Family-level decision-making for genetic research

The study addressed the pattern of decision making in the context of the social stigma borne by podoconiosis-affected families. In genetic studies, family members of participants may indirectly become part of a genetic study without their consent and the results of the study may affect the whole family [11]. This issue was critical in our study because stigma was attached to the whole family of patients and because patients considered that stigma might intensify because of the study. As a result, participants insisted that the appropriateness of the study should be evaluated at the level of the family before seeking consent from the individual. Families needed to be given appropriate information and sufficient time to discuss it amongst themselves before individual participants were approached to enrol into the research. These findings corroborated others' that argue that the individual-based informed consent model does not fit genetic studies in which the family is the unit of analysis [9,10]. Including families in the consent process of genetic family studies is essential to ensure that informed decisions are made in such studies [27] and that complex intra-household decision-making dynamics are respected as appropriate [28].

It should be understood, however, that familial approval to participate in genetic research is preferred not because individual decision-making is not the norm in Wolaita like some African traditional communities [21]. People in Wolaita insist that the right of any competent member of a family to refuse participation in genetic research should be respected, indicating the widely supported view that group-level approval, though desirable, cannot be a proxy for individual consent [1,7,8,29-33].

### The actual design and conduct of the consent process

Our findings highlighted the need to design a consent process that ensured that participants were not further stigmatized and that explained the genetic component of the research without undermining the established relationship of MFTPA with patients. Therefore, we developed a strategy aimed at reaching consensus (i) with the MFTPA over the design of a consent process to support participants' comprehension of the purpose of the genetic study; and (ii) with fieldworkers and the community about the strategies available to combat further stigmatization as a result of the study.

We started by training MFTPA fieldworkers on the development of podoconiosis among genetically susceptible people exposed to red clay soils. The training emphasized that people protected from the red soils were unlikely to develop disease even if they were genetically susceptible. In addition, we emphasized the effectiveness of simple interventions like foot wear and hygiene. We illustrated the discussion using the example of podoconiosis previously being common in countries of North Africa (Algeria, Tunisia, Morocco and the Canary Islands) and Europe (France, Ireland and Scotland), but now being eradicated through the universal use of shoes [20]. We explained that people from these populations may still have the genetic factor that makes them susceptible, and might develop the disease if they were exposed. In addition, we mentioned that non-symptomatic people in the community may also carry the genetic factor. This simplified explanation of the mixed role of both genetic and environmental factors was understood by the fieldworkers.

Following this, we conducted community discussion fora on issues related to the familial nature of podoconiosis, about which there are a number of rumours which are rarely discussed in public. Administrators and fieldworkers of MFTPA held frequent discussions with the podoconiosis community during routine outreach clinic visits and discussed the role of familial and environmental factors in the causation of the disease. Every discussion was focused on the effectiveness of footwear and good personal hygiene both to prevent and to treat the early stages of podoconiosis [34].

The process was enriched by suggestions from patients that the education should also embrace non-affected members of the community. Podoconiosis affected people stressed that non-affected people should be informed that the disease is preventable by wearing shoes even in the presence of familial factors. As a result, we conducted expanded health education programs to improve the awareness of the unaffected community and health professionals. An important aspect of the task was to reduce stigma experienced by podoconiosis affected people and their families. After the community sensitization and feedback, the MFTPA started to openly discuss the possible heritability of the disease. In addition, it started recruiting non-symptomatic children of affected families as targets for preventive intervention.

Future plans include dissemination of the findings of the genetic research to both affected and non-affected members of the community. In addition, via the MFTPA, we will continue to educate the whole community and stress universal use of footwear to avoid a false sense of security among currently unaffected families who may also be genetically susceptible. Examples could be gained from experience of community engagement strategies in Kilifi, Kenya [35].

The strategy we used combined information on the conduct of research with provision of health education to both patients and the presumed stigmatizing population and yielded very satisfactory results. Our discussions with the community and family appeared effective in diminishing the perceived risk of many people. Although we did not formally evaluate the intervention, most participant families indicated they no longer had concerns over stigma during subsequent recruitment by fieldworkers. Of more than 200 families approached, only three denied that patients with clear signs of disease were affected. In-depth inquiry by the research nurses revealed that the daughters concealed their legs so that people did not know they were patients. Their parents were afraid that participation could disclose this secret and were not convinced by the strategy available for keeping confidentiality. We also ensured that we had satisfactory collaborative partnership with the MFTPA without threatening the trust it had built with the community [36].

## Conclusion

The study showed that investigators must seek to assess and address risks of research from prospective participants' perspectives. This involves understanding the issues in the society, the culture, community dialogues and developing a consent process that takes all these into consideration. The rapid assessment enabled us to design an appropriate and locally acceptable consent process for genetic research which had the potential to aggravate stigmatization experienced by patients. We also observed that the interventions linked with the consent process facilitated recruitment of participants and increased their confidence that the research would not fuel stigma. In addition, the interventions enabled informed community discussion of a topic previously subject to rumour and supported MFPTA staff's ability to discuss familial aspects of the study on an ongoing basis. The genetic study did not have immediate therapeutic benefit to the study participants, however, the rapid assessment helped bridge the communication gap among unaffected individuals in the community, affected families and the MFTPA in the effort to prevent and treat the disease. The previous study on the genetics of podoconiosis has also strengthened the rationale for providing shoes for unaffected children of affected families [12].

The results of the study are applicable to other stigmatizing genetic diseases with or without effective treatment. Our findings show that the most important strategy in conducting genetic research in such instances is appropriately addressing the social influences, personal preferences and felt risks through counselling and education prior to the conduct of the actual study. It also showed that researchers should study and respect the decision making pattern of a community and a family before approaching individual participants for a genetic study. We believe the findings have practical uses for biomedical investigators and others interested in the consent process of studies conducted in socially stigmatized diseases and in research assumed to bear the risk of fuelling stigma among groups.

## Competing interests

All authors declare that they have no conflict of interest associated with the publication of this manuscript.

## Authors' contributions

FT collected data in the field, drafted the paper, and designed the study in conjunction with SB, BF, MN, AA, CR and GD. FT developed the study instruments with assistance from BF and SB. FT performed the analysis assisted by SB. MN, GD, AA and CR obtained funding. GD supervised the field work. All authors interpreted data and critically revised the paper for substantial intellectual content, and read and approved the final manuscript.

## Pre-publication history

The pre-publication history for this paper can be accessed here:


